# Seeking pleasure or seeking compensation: a dual-pathway model of problematic social networks use

**DOI:** 10.1016/j.abrep.2026.100720

**Published:** 2026-06-19

**Authors:** Lena Klein, Matthias Brand, Elisa Wegmann

**Affiliations:** a Department of General Psychology: Cognition, Faculty of Computer Science, University of Duisburg-Essen, Duisburg, Germany; b Center for Behavioral Addiction Research (CeBAR), Center for Translational Neuro- and Behavioral Sciences (C-TNBS), University Hospital Essen, University of Duisburg, Essen, Germany; c Erwin L. Hahn Institute for Magnet Resonance Imaging, Essen, Germany

**Keywords:** Problematic social media use, Motives, Personality, Reinforcement mechanisms, Coping, Gratification, Screen time

## Abstract

Social networks (SN) are used for different reasons in everyday life, often resulting in higher usage time and sometimes in a problematic use of social networks (PSNU). The I-PACE model outlines that specific predisposing variables (e.g., usage motives, personality traits, stress-appraisal, coping strategies) and affective and cognitive mechanisms (e.g., use expectancies, gratification, compensation) as well as their interactions contribute to the development and maintenance of PSNU. Our aim was to test a structural equation model with two different paths of reinforcement mechanisms: pleasure-seeking for achieving specific emotional experiences and compensation-seeking to avoid or reduce negative states. Both pathways are assumed to lead to higher symptom severity of PSNU and usage time of SN. In a pre-registered, multi-center study (DFG-funded research unit) with a mixed-method, cross-sectional, between-subject design in Germany, 306 (81% female; Age: *M* = 26.41 years; *SD* = 07.76, Range: 16–64 years) participants underwent the study procedure, including a diagnostic interview based on DSM-5 criteria for gaming disorder (modified for PSNU), thereby identifying individuals with problematic use of SN. Additional constructs were assessed by self-reports. The structural equation model provided an acceptable fit with the data. It showed that positive reinforcement mediates the effect of usage motives on SN usage time. Negative reinforcement mediates the effect of stress-appraisal and coping on PSNU and the effect of usage motives on PSNU. The pleasure-seeking pathway significantly leads to higher usage time of SN while the compensation-seeking pathway results in higher symptom severity. The results emphasize the relevance of different predisposing factors and reinforcement processes, and expands existing theoretical models.

## Highlights

• We tested a two-pathway model of problematic social network use (PSNU).
• A sample of 306 participants with pathological, risky, and non-problematic use.
• Usage motives of social networks played an important role in both pathways.
• Positive reinforcement leads towards higher usage time of social networks.
• Negative reinforcement leads towards higher symptom severity of PSNU.

## 1 Introduction

5.24 billion people use social networks (SN) every day for multiple reasons (Statista, 2025). In our digitalized society, these applications could be used anywhere and anytime. This results in higher usage times, for example, 48% of people aged 18–25 stated that they use SN between 6 and 20 h a week and 11% stated, that they exceeded 20 h (Statista, 2024). For some people, the increasing usage of SN could lead to a loss of control over the usage behavior and to negative consequences in daily life resulting in problematic social networks use (PSNU; Wegmann & Brand, 2019). There is discussion about the extent to which usage time plays a role in PSNU (Rozgonjuk et al., 2020). While some researchers assume that usage time has a negative impact on mental health (Twenge & Farley, 2021), other studies show that there is no direct influence of usage time, but rather that this is mediated by specific variables, such as psychopathology (Rozgonjuk et al., 2020). With the inclusion of gaming disorder as “disorder due to addictive behaviors” in the ICD-11 (World Health Organization, 2022), it is discussed whether PSNU should be considered as a distinct disorder with specified symptoms under the category “other specified disorders due to addictive behavior” (6C5Y). However, until now, there is no official classification for PSNU in the ICD-11 and it is also not mentioned in the DSM-5 (Fineberg et al., 2022; Moretta & Wegmann, 2025). In literature there are different conceptual and terminological approaches, for example social networks use disorder (Rumpf et al., 2025), but since there is no official classification yet, we will use the term PSNU instead of labeling it as a disorder.

Despite there being no PSNU diagnosis in the official manuals, authors have proposed theoretical models to explain PSNU that include factors such as personality, coping, usage motivation, loneliness, reinforcement processes. For example, the Interaction of Person-Affect-Cognition-Execution (I-PACE) model (Brand et al., 2019) describes the interplay of predisposing variables with affective and cognitive mechanisms, which could result in the development and maintenance of problematic usage of the Internet. The model distinguishes between predisposing factors that contribute to a problematic usage of the Internet in general and application-specific predisposing factors such as PSNU. General predisposing factors (e.g., stress vulnerability, personality) may lead to problematic use of the Internet regardless of the specific application model. Specific predisposing factors (e.g., usage motives, social integration) are characteristic of certain applications, making individuals more likely to use a specific application in a problematic way (Brand et al., 2019). Both types of predisposing factors interact with reinforcement mechanisms considered as driving factors in the addiction process (Brand et al., 2025). There is a distinction made between positive reinforcement that increases the probability of performing a specific behavior if it has been previously been rewarded, and negative reinforcement, if a negative state (e.g., negative mood or stress) was previously removed or reduced through the specific behavior (Wegmann et al., 2022).

In a narrative review, Wegmann and Brand (2019) discuss a theoretical two-pathway model for the development and maintenance of PSNU as a specification of the I-PACE model and further theoretical models explaining media usage behavior (e.g., Uses and Gratification Theory (Katz et al., 1973; Rubin, 1993), applied to SN. The first path is the fear-driven/compensation-seeking hypothesis, in which the predisposing variables such as social anxiety, depression, and loneliness interact with negative reinforcement processes. The reward-driven hypothesis consists of predisposing variables like narcissism, and specific needs (e.g. need to belong, need for popularity), which interact with positive reinforcement processes. Wegmann and Brand (2019) point out that these two pathways could work as a reinforcement circle and interact with each other leading to PSNU (Brand, 2022; Wegmann & Brand, 2019). In the current interpretation of the I-PACE model it is argued that even if a behavior is motivated by the anticipation of certain compensatory effects, gratification may be experienced as well (Brand et al., 2025). The role of usage time as a predictor of PSNU is still unclear (Burén et al., 2023), but the literature is indicating, that even if it does not serve as a symptom of PSNU, it could be an indicator for an intense usage, and is more closely associated with reward-related motivations than PSNU(Wadsley et al., 2022). Together, the models argue that PSNU develops when predisposing traits and needs lead to strong motivational drivers, which are reinforced through either gratification or relief from negative feelings, increasing the risk of problematic use. According to Brand (2022), positive reinforcement works at first as a driver for engaging in the behavior, which could lead to higher usage time, as well as to problematic behavior, whereas negative reinforcement later on becomes part of the problematic use as the behavior becomes more frequently manifested. It is possible that certain pathways lead to more intense use of SN with higher usage time, but result in less problematic behavior – and vice versa.

While the I-PACE model encompasses a broad range of predisposing factors, the present study concentrates on a theory-driven selection of both general and SN-specific predisposing variables. We particularly focus on factors that serve as motivational drivers for using SN, as these are assumed to interact closely with the affective and cognitive mechanisms underlying PSNU. The study also considers the two-path model of Wegmann and Brand (2019) and the interaction with positive and negative reinforcement processes. In addition, we aim to investigate what leads to higher usage time of SN and what leads to higher symptom severity, as it is not yet fully understood how these mechanisms are related. To our knowledge, this is the first attempt to empirically test the model by Wegmann and Brand (2019) with two pathways leading to PSNU and/or usage time.

Addressing the effect of general predisposing factors, we defined “personality-related predisposing variable” and “stress-appraisal & coping” as latent dimensions including personality traits, stress vulnerability, self-directedness, and coping styles. In a meta-analysis Fioravanti et al. (2025) showed, that there are some psychological risk factors specifically for PSNU and some factors shared with other forms of problematic use of the Internet. In this model we focused on more SN-specific factors or factors, that are underrepresented in the literature, supported by this meta-analysis. The dimension “personality-related predisposing variable” is defined by extraversion and narcissism, because these personality traits reflect a certain need for self-presentation and both showed a significant association with PSNU and usage time (Barry & McDougall, 2018; Casale & Banchi, 2020; Kocak et al., 2020; Kuss & Griffiths, 2011; Wegmann & Brand, 2019). The dimension “stress-appraisal & coping” is defined by coping styles and self-directedness, whereby maladaptive coping styles and stress vulnerability are associated with a higher tendency towards PSNU (Lazarus & Folkmann, 1984; Moretta et al., 2023; Sriwilai & Charoensukmongkol, 2016; Wegmann & Brand, 2016). Self-directedness is defined as the ability to perceive oneself as an autonomous self and to influence situations in a self-determined way in accordance with one's goals and values (Celikel et al., 2009) and is shown as a predisposing variable in the feels-better-pathway, based on the I-PACE model as a driver of problematic internet use (Brand et al., 2026).

Behavior-specific predisposing variables are defined by the I-PACE model as specific needs and motives (Brand et al., 2019). We defined “motivational SN-related predisposing variable” and “loneliness-related SN predisposing variable” as latent dimensions. It has been empirically shown that certain usage motives (e.g. information seeking, personal utility and altruistic motives) could lead to a higher risk of PSNU (Al-Menayes, 2015; Kircaburun et al., 2020). Furthermore, loneliness, often seen as an indicator for mental health problems (Zhang et al., 2022), is discussed as a specific risk factor for PSNU. A meta-analysis showed, that overall, there is a positive correlation between SN usage and loneliness (Zhang et al., 2022).

**Fig. 1:**
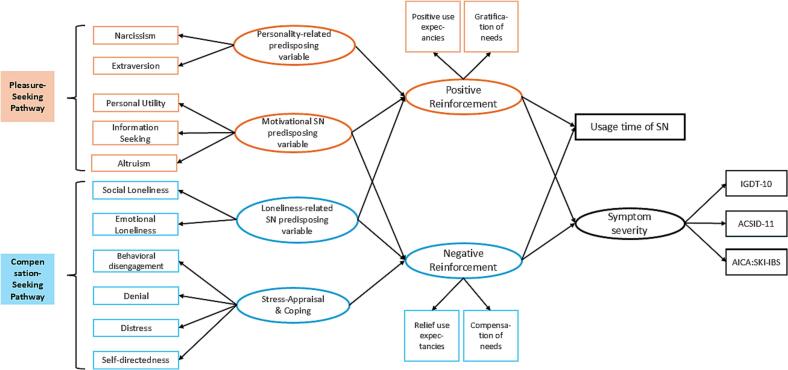
Structural equation model as preregistered. Orange represents the pleasure-seeking pathway, while blue represents the compensation-seeking pathway. Symptom severity is measured by the Internet gaming Disorder Test 10 (IGDT-10), Assessment of Criteria for Specific Internet-Use Disorders (ACSID-11) and the structured diagnostic interview (AICA:SKI-IBS). (For interpretation of the references to colour in this figure legend, the reader is referred to the web version of this article.)

Considering these predisposing variables against the background of the model assumptions by Wegmann and Brand (2019) reveals that different mediation effects of positive and negative reinforcement processes are postulated (Fig. 1): pleasure-seeking pathway and compensation-seeking pathway. The mediator variable positive reinforcement is represented by positive use expectancies and gratification of needs through the usage. Negative reinforcement consists of negative use expectancies and compensation of needs. Symptom severity is defined as latent dimension by standardized questionnaires and a diagnostic interview considering DSM-5 and ICD-11 criteria of (Internet) gaming disorder, modified for PSNU.

For the pleasure-seeking pathway (upper path in Fig. 1), it is hypothesized that “personality-related predisposing variables” and “motivational SN-predisposing variables” have an effect on usage time and symptom severity mediated by positive reinforcement. Moreover, the effect of “motivational SN-predisposing variables” on usage time and symptom severity is mediated by negative reinforcement. It is expected that the pleasure-seeking pathway leads to higher usage time of SN rather than to higher symptom severity.

For the compensation-seeking pathway (lower path in Fig. 1), it is hypothesized that the effect of “stress-appraisal & coping” on usage time and symptom severity is mediated by negative reinforcement, while the effect of “loneliness-related SN predisposing variable” on usage time and symptom severity is mediated by both, positive and negative reinforcement. It is assumed that this pathway will lead more to the symptom severity, than to higher usage times of SN.

## 2 Method

### 2.1 Participants and study design

The sample of this study consists of participants recruited in a multi-center study funded by the German Research Foundation (FOR2974; for details see preregistration on Open Science Framework: https://osf.io/n5cd7). The data from two subprojects (OSF: https://osf.io/ehq98; https://osf.io/9j8g3) were used. Data collection was carried out between December 2021 and August 2024, at multiple sites in Germany (University of Duisburg-Essen, University of Lübeck, Hannover Medical School). The projects recruited via mailing lists, local advertisement, and social media. Interested participants underwent a standardized telephone screening based on DSM-5 criteria for gaming disorder (AICA-C9) and modified for the specific target behaviors, as well as the check of exclusion and inclusion criteria (https://osf.io/k62zc) and a potential group assignment. For the following results only the participants included for behavior social networks use will be considered. The study consists of a random sample drawn from the general population in order to include individuals with problematic use but also individuals with non-problematic or risky use. Initially, there were 310 participants in the data set, but during data cleansing two participants were excluded due to missing data and further two participants were excluded because of unrealistic usage times of social networks (more than 15 h on a weekday). Overall, 306 participants (249 females; 55 males; 2 nonbinary) aged between 16 and 64 years (*M* = 26.41 years; *SD* = 07.76) have been included. Most of the participants were students (64%), followed by employees (15%), part-time employees (11%), and other categories. The mean usage time of SN per workday was *M* = 237.84 min (*SD* = 114.61; Range: 35.00–750.00).

#### 2.1.1 Ethics

For the entire study protocol, approval was given by the local ethics committee of the University of Duisburg-Essen (ID: 1911APBM0457), Hannover Medical School (ID: 8767_BO_S_2019) and the University of Lübeck (ID: 20-081) in Germany. All participants were informed about the study, gave voluntary informed consent, and were debriefed at the end of the laboratory research.

### 2.2 Measures

All the measures and detailed descriptions of the questionnaires could also be looked up in the pre-registration on OSF (https://osf.io/k62zc; Date of publication: May 2025) and the supplementary material for the calculation. The reliability of the scores is reported in Table 1. For the specific position of the variables in the SEM, please see Fig. 1.

**Table 1:** Descriptive values*.*

	*M*	*SD*	Min	Max	Skewness	Kurtosis	Cronbach's alpha
AICA:IBS (Diagnostic interview, symptom severity)	03.47	03.01	00.00	09.00	00.17	−01.40	n.a.
ACSID-11 frequency (symptom severity)	14.88	08.62	00.00	33.00	00.24	−00.74	0.93
IGDT-10 (symptom severity)	02.35	02.44	00.00	09.00	00.93	00.00	0.86
Usage time of SN	237.84	114.61	35.00	750.00	01.07	02.18	n.a.
Gratification of needs (EGS; Positive Reinforcement)	01.38	00.75	00.00	04.00	00.63	00.74	0.66
Compensation of needs (ECS; Negative Reinforcement)	01.35	00.84	00.00	03.67	00.41	−00.33	0.71
Positive use expectancies (IUES, Positive Reinforcement)	04.23	00.86	01.50	06.00	−00.44	00.44	0.82
Relief use expectancies (IUES; Negative Reinforcement)	04.01	01.19	01.00	06.00	−00.51	−00.23	0.76
Extraversion (BFI-2; Personality)	03.32	00.68	01.50	04.92	−00.16	−00.40	0.85
Narcissism (NN; Personality)	04.24	02.21	01.00	09.00	00.27	−01.06	0.88
Personal Utility (MUSM; Motives)	02.89	00.77	01.00	05.00	−00.04	−00.24	0.56
Information Seeking (MUSM; Motives)	03.84	00.73	01.75	05.00	−00.50	−00.28	0.58
Altruism (MUSM; Motives)	02.16	00.88	01.00	05.00	00.53	−00.38	0.58
Social Loneliness (LON; Loneliness)	01.37	01.06	00.00	03.00	00.64	−01.18	0.85
Emotional Loneliness (LON; Loneliness)	01.00	01.17	00.00	03.00	00.19	−01.19	0.68
Behavioral Disengagement (COPE; Coping)	01.55	00.56	01.00	03.50	00.96	00.58	0.61
Denial (COPE; Coping)	01.69	00.69	01.00	04.00	01.00	00.46	0.48
Distress (TICS; Stress-Appraisal)	21.68	09.09	02.00	44.00	00.02	−00.44	0.86
Self-Directedness (TCI; Stress-Appraisal)	29.44	07.85	06.00	44.00	00.14	−00.43	0.88

*Note*. *N* = 306; To test the reliability of scores only consisting of two items (Altruism (MUSM; motives); Behavioral Disengagement and Denial (COPE; Coping)), Spearman-Brown was used.

**Personality-related predisposing variable**: For this latent variable of personality traits, we used the subscale *extraversion* of the German Big Five Inventory-2 (Danner et al., 2019; Soto & John, 2017). Secondly, we used the German version of the Naughty Nine Scale (Küfner et al., 2015) for assessing the dark tetrad personality traits like *narcissism*. In both scales, higher cores represent a stronger manifestation of the personality trait.

**Motivational SNS predisposing variable**: To assess the different usage motives, we used the Motives for Using Social Media Scale (MUSM; (Al-Menayes, 2015)) and translated it in German by back-and-forth translation. We focused on *personal utility*, *information seeking,* and *altruism*.

**Loneliness-related SNS predisposing variable**: The subscales *emotional loneliness* and *social loneliness* of the short Loneliness Scale (LON; (Gierveld & Tilburg, 2006)), translated in German, were used. Higher scores indicating higher levels of loneliness.

**Stress-Appraisal & Coping**: This latent variable consists of three different scales. The subscales maladaptive coping strategies *denial* and *behavioral disengagement* of the Brief COPE (Carver, 1997; Knoll et al., 2005). The second manifest variable measures general stress vulnerability by using the German version of Trier Inventory of Chronic Stress (TICS; (Schulz et al., 2004)) subscale *chronic stress screening scale*. The third questionnaire is the Temperament and Character Inventory (TCI; (Cloninger et al., 1994; Petrowski et al., 2012)) for assessing self-directedness. In all scales, higher scores indicating higher expression of coping, chronic stress or self-directedness.

**Positive Reinforcement**: To measure gratification, we used the sub facet *gratification of needs* of the Experience of Gratification Scale (EGS; (Wegmann et al., 2022)). As an additional part of the reinforcement processes, we included positive use expectancies on SN. Therefore, we used the subscales *positive expectancies* of the Internet Use Expectancies Scale modified for SN use (IUES; (Brand et al., 2014)). Both scales were validated in German and higher values indicate higher expectancies or gratification/compensation.

**Negative Reinforcement:** The experienced *compensation of needs* was measured by Experience of Compensation scale (ECS; (Wegmann et al., 2022)). Additionally, we used the subscale *relief expectancies* of the IUES (Brand et al., 2014).

**Symptom severity** has been measured by using self-reports and a structured diagnostic interview, both together covering DSM-5 criteria and ICD-11 criteria, all modified for PSNU. To ensure that all participants had the same understanding of SN, a brief definition was provided at the start, along with examples of the platforms and the manner in which they are used. We used the Internet Gaming Disorder Test-10 (IGDT-10; (Király et al., 2017) translated in German language) in this study for assessing the DSM-5 criteria. The Assessment of Criteria for Specific Internet-Use Disorders (ACSID-11; (Müller et al., 2022)) is based on the ICD-11 assessment criteria for different types of problematic usage of the Internet and validated in German language. Only responses relating to SN use were taken into consideration. For both scales cut-offs were possible and described in the Supplementary material. The structured diagnostic interview, the AICA-SKI:IBS (Müller et al., 2017) in its German version, was used to assess problematic usage of the Internet based on the DSM-5 criteria. Interviews were conducted by trained doctoral students, who received clinical-diagnostic training and regular supervision by experienced clinicians. With this interview, individuals could be classified into certain groups: non-problematic (up to one criterion fulfilled; *n* *=* *112*), risky use (< four criteria fulfilled, *n* = 72) and PSNU (five to nine criteria fulfilled; *n* = 123). For the SEM, we used a sum-score of the fulfilled criteria.

The **usage time of social networks** was assessed at the end of the study. Participants were asked how long they spend on SN on a typical workday (calculated in minutes), because it reflects their everyday life the best.

### 2.3 Statistical analyses

Statistical analyses were carried out in IBM SPSS 29.0.0 for Windows to analyze descriptive statistics and correlational analyses. Pearson correlations were calculated for testing bivariate relationships between two variables (Cohen et al., 2003). For analyzing the mediation and path analysis, we used Structural Equation Modelling (SEM) at the latent level with MPlus 8.3 (Muthén & Muthén, 2011). We evaluated the model fit with the standard criteria: standardized root mean square residual (SRMR; values <0.08 indicate a good fit), root mean square error of approximation (RMSEA; values <0.08 indicate a good and 0.08–0.10 an acceptable model fit) and comparative fit indices (CFI/TLI; values >0.90 indicate an acceptable fit) (Hu & Bentler, 1995; Hu & Bentler, 1998). The SEM analysis was conduced using the Maximum Likelihood (LM) estimator and indirect effects were calculated using the Delta-Method, the default approach in MPlus. A post hoc power analysis showed that, according to the rule of thumb N:q ratio of at least 5:1 (Bentler & Chou, 1987), for the modified model, we need a 5:77 parameters, which results in a sample size of 385. While the available sample size falls below this recommendation, Wolf et al. (2013) has shown that SEMs can yield reliable estimates with smaller sample sizes, provided the data quality is high and the model is well-specified, which is given through the laboratory setting and hypothesis-driven model and our model fit is good.

## 3 Results

### 3.1 Descriptive values and correlation analyses

The descriptive values as mean scores and standard deviation of all scales are shown in Table 1. The bivariate correlations between all the variables are shown in Table 2. It is shown that the different scores for symptom severity correlate with most of the other scales with small to medium effect sizes. The usage time, as other dependent variable, also shows significant correlations with most of the questionnaires.

**Table 2:** Pearson correlations*.*

	1	2	3	4	5	6	7	8	9	10	11	12	13	14	15	16	17	18
1. AICA:IBS (Diagnostic interview; symptom severity)	–																	
2. ACSID-11 frequency (symptom severity)	0.739**	–																
3. IGDT-10 (symptom severity)	0.676**	0.783**	–															
4. Usage time of social networks	0.382**	0.372**	0.450**	–														
5. Gratification of needs (EGS; Positive Reinforcement)	0.219**	0.174**	0.194**	0.152**	–													
6. Compensation of needs (ECS; Negative Reinforcement)	0.452**	0.405**	0.410**	0.221**	0.534**	–												
7. Positive use expectancies (IUES; Positive Reinforcement)	0.251**	0.204**	0.285**	0.298**	0.426**	0.365**	–											
8. Relief use expectancies (IUES; Negative Reinforcement)	0.544**	0.561**	0.533**	0.342**	0.259**	0.556**	0.360**	–										
9. Extraversion (BFI-2; Personality)	−0.149**	−0.118*	−0.051	−0.125*	0.061	−0.106	−0.071	−0.154*	–									
10. Narcissism (NN; Personality	0.185**	0.137*	0.191**	0.115*	0.206**	0.244**	0.178**	0.209**	0.156**	–								
11. Personal Utility (MUSM; Motives)	0.137*	0.105	0.174**	0.225**	0.454**	0.265**	0.320**	0.115*	0.031	0.108	–							
12. Information Seeking (MUSM; Motives)	0.116*	0.098	0.121*	0.107	0.238**	0.162**	0.170**	0.071	0.022	−0.055	0.378**	–						
13. Altruism (MUSM; Motives)	0.082	0.073	0.142*	0.128*	0.317**	0.243**	0.222**	0.059	0.032	−0.061	0.538**	0.349**	–					
14. Emotional Loneliness (LON; Loneliness)	0.251**	0.238**	0.257**	0.191**	0.110	0.323**	0.150**	0.330**	−0.295**	0.202**	0.019	−0.008	0.017	–				
15. Social Loneliness (LON; Loneliness)	0.166**	0.134*	0.122*	0.085	0.127*	0.233**	0.096	0.162**	−0.257**	0.082	0.051	0.026	0.037	0.408**	–			
16. Behavioral Disengagement (COPE; Coping)	0.224**	0.228**	0.185**	0.230**	0.148**	0.211**	0.096	0.277**	−0.159**	0.124*	0.040	−0.080	0.039	0.261**	0.160**	–		
17. Denial (COPE; Coping)	0.247**	0.285**	0.275**	0.134*	0.190**	0.287**	0.088	0.325**	−0.013	0.170**	0.122*	0.064	0.207**	0.203**	0.091	0.348**	–	
18. Distress (TICS; Stress-Appraisal)	0.328**	0.372**	0.384**	0.123*	0.122*	0.310**	0.137*	0.383**	−0.214**	0.245**	0.064	0.072	0.107	0.491**	0.275**	0.249**	0.334**	–
19. Self-Directedness (TCI; Stress-Appraisal)	−0.334**	−0.334**	−0.317**	−0.265**	−0.064	−0.261**	−0.014	−0.412**	0.413**	−0.271**	−0.005	−0.036	0.031	−0.508**	−0.375**	−0.397**	−0.251**	−0.573**

*Note. N* = 306; ** *p* < 0.01., * *p* < 0.05.

### 3.2 The structural equation model

**Fig. 2:**
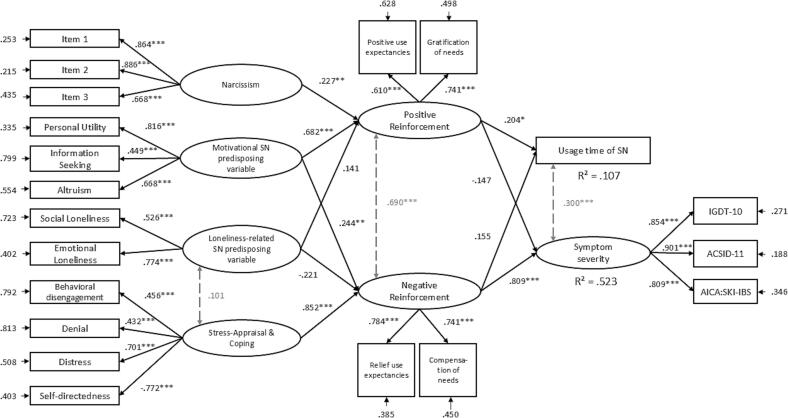
Final structural equation model results; *p < 0.05; **p < 0.01; ***p < 0.001; Symptom severity is measured by the Internet Gaming Disorder Test 10 (IGDT-10), Assessment of Criteria for Specific Internet-Use Disorders (ACSID-11) and the structured diagnostic interview (AICA-SKI:IBS).

In the preregistered SEM, two paths were assumed. The fit of the SEM indicated an acceptable, but not ideal fit with the data (RMSEA = 0.067 with *p* < 0.002, CFI = 0.904, TLI = 0.879, and SRMR = 0.060; χ2 = 321.30, *p* < 0.001, χ2/df = 2.36). We defined an alternative model and excluded extraversion which did not correlate with all the scales and did not fulfil the criteria for mediation analysis (Baron & Kenny, 1986). With this adjustment, the model showed an acceptable to good fit to the data (RMSEA = 0.051 with *p* < 0.394, CFI = 0.946, TLI = 0.933, and SRMR = 0.048; χ2 = 277.10, *p* < 0.001, χ2/df = 1.81). No further modifications were made, as the fit indices were already within an acceptable range, all paths are theoretically justified, and additional adjustments would only result in marginal improvements in the fit at the expense of weaker theoretical justification. Symptom severity (*R*^2^ = 0.523, SE = 0.060, *p* < 0.001) and usage time of SN (*R*^2^ = 0.107, SE = 0.038, *p* = 0.005) were significantly predicted by the proposed variables. The whole structural equation model with the factor loadings and the β-weights are shown in Fig. 2.

The results indicate that all latent dimensions are well-represented by the manifest variables. Moreover, negative reinforcement significantly predicted symptom severity of PSNU, whereas positive reinforcement did not. In contrast, positive reinforcement showed a direct effect on the usage time of SN, while negative reinforcement did not. Narcissism and the motivational SN predisposing variable had significant direct effects on positive reinforcement. The motivational SN predisposing variable and stress-vulnerability and coping have a significant effect on negative reinforcement. The loneliness-related SN predisposing variable did not affect positive nor negative reinforcement significantly.

Analyzing the mediation effects predicting usage time, the results illustrate that the effect of motivational SN predisposing variable on usage time of SN was significantly mediated by positive reinforcement (β = 0.139, *SE* = 0.066, *p* = 0.036). There were no further mediation effects predicting usage time by positive reinforcement: narcissism (β = −0.046, *SE* = 0.025, *p* = 0.066), loneliness (β = 0.029, *SE* = 0.022, *p* = 0.195). Regarding symptom severity, motivational SN predisposing variable on symptom severity were significantly mediated by negative reinforcement (β = 0.198, *SE* = 0.066, *p* = 0.003). The effect from stress-appraisal & coping on symptom severity was also significantly mediated by negative reinforcement (β = 0.689, *SE* = 0.163, *p* < 0.001).

## 4 Discussion

Overall, the results illustrate that the effects of general and specific predisposing factors on symptom severity of PSNU and usage time are significantly mediated by positive and negative reinforcement processes. This supports the assumption by Wegmann and Brand (2019) emphasizing two different pathways: pleasure-seeking and compensation-seeking. Furthermore, relevance of differentiation between general and specific predisposing factors and their contribution to the development and maintenance of a problematic behavior as suggested by the I-PACE model (Brand et al., 2019) is highlighted.

The study also identifies potentially different processes in predicting intensive, but non-problematic use, and problematic use. Usage time is predicted by usage motives mediated by positive reinforcement. Using SN to fulfill motives like seeking information, joining groups for conversations or helping people are related to positive experiences and expectancies resulting in higher usage times. Interestingly, positive reinforcement does not lead to higher symptom severity, which is largely in line with our assumption of a small effect. Nevertheless, considering usage time as indicator of an intense but not per se problematic use in distinction from an online behavior associated with subjective impairments expands the model of Wegmann and Brand (2019), and also the I-PACE model.

We also found significant effects of usage motives and stress-appraisal and coping on symptom severity mediated by negative reinforcement. It illustrates that certain usage motives are related to suppress negative feelings and escape reality through SN usage. Furthermore, it indicates that individuals who are more vulnerable to stress, seem to be less self-directed and tend to cope in maladaptive ways, are at higher risk using SN to feel less bad or anxious and to avoid problems. These findings are supported by various studies that have demonstrated the effect of coping strategies as an indicator of a problematic behavior (Moretta et al., 2023; Sriwilai & Charoensukmongkol, 2016). According to Kessling et al. (2025), also acute stress causes to feel a subjective urge to use social networks. Therefore, considering the interplay of general stress vulnerability, acute stress as well as maladaptive coping strategies, it might be an indicator that if individuals try to compensate their needs and higher stress with using SN it could be associated with a higher risk to develop a tendency towards PSNU.

Contrary to our assumption, loneliness did not predict the pleasure-seeking nor the compensation-seeking pathway. However, SN have presented a mixed picture on the significance of loneliness: a meta-analysis by Zhang et al. (2022) found that the relationship between loneliness and SN use depends on the usage type. Their results suggest that the type of SN use could be an important additional factor when considering loneliness. There were no mediated effects of narcissism on either usage time or symptom severity. However, narcissistic traits show a predictive effect on the rewarding, feel-successful path on positive reinforcement. The relationship between narcissism and symptom severity is in line with previous studies (Casale & Banchi, 2020). Balcerowska et al. (2023) also concluded that this relationship is rather mediated by negative expectancies, thereby it could be assumed that narcissism could affect symptom severity in later stages of PSNU development, when more negative consequences in combination with more negative emotion regulation are felt.

Overall, we could highlight the interplay of predisposing variables and reinforcement processes resulting in PSNU assumed in the I-PACE model. Thereby, we contribute to the distinction between general and specific assumptions explicitly for SN. Furthermore, we focused on explicit hypotheses to align the inner circle's reinforcement cycle. The results support the assumption of two different pathways of positive and negative reinforcement processes leading to either higher usage time of SN and symptom severity of PSNU. Based on current interpretations of the I-PACE model, it could be assumed that there is a wave-like progression of the two reinforcement mechanisms (Brand et al., 2025). Our results illustrate that positive reinforcement significantly could predict usage time, while negative reinforcement seems to be more related to higher symptom severity. The results also support the structure of a reward-driven- and fear-driven-pathway suggested by Wegmann and Brand (2019) and expanded by systematically differentiation between symptom severity and usage time as indicator of an intense, but not problematic online behavior. Future studies should be more selective in the identification of predisposing variables (e.g., specific usage motives, personality characteristics, social integration, and Fear of Missing Out) related to an intense or problematic behavior, or both. Moreover, how the development and dominance of positive and negative reinforcement mechanisms evolve and possibly also change over time as well as in the course of behavioral manifestation providing further insights into mechanisms of causality and stability of time should be examined.

Nevertheless, limitations must be mentioned. This study examines specific predisposing variables as exemplary cases. Future studies should systematically expand on the relevance of general and specific characteristics, also against the backdrop of a potential conceptualization of the disorder (Moretta & Wegmann, 2025). Additionally, we investigated the use of SN platforms in general and did not differentiate between different applications in detail. Especially, when considering usage motives, applications are used for various purposes (Kircaburun et al., 2020), such as communication via messages or entertainment through short videos. Moreover, we used cross-sectional data and cannot assume causality. In addition, the sample size calculated in the power analysis using a post hoc test was not achieved. Nevertheless, our model fit was good and the data quality was high. Future studies should examine the development of usage and symptom severity as well as the interaction and change of predisposing variables and reinforcement mechanisms. Thereby, it might be worth including objective data regarding usage time in order to obtain a better overall picture of usage, intensity, and also the potential strain in everyday life (Rozgonjuk et al., 2018; Ryding & Kuss, 2020). The gender distribution is also not balanced, as there is a female dominance in the data which is based on the fact that one subproject focused on women solely. A generalization is not possible, even if previous studies already illustrated that specific reinforcement processes are relevant in genders (Wegmann et al., 2017).

## 5 Conclusion

Access to social networks is ubiquitous, easy, and has become established even in everyday social situations. This could result in intense, but non-problematic use. This study illustrates that usage time is not necessarily linked to the same constructs as symptom severity. Social network use needs to be considered in a more differentiated way to provide a better understanding of intensity as well as the conceptualization of a potential problematic behavior.

This study also provides further insights into better understanding of the underlying mechanisms of PSNU and could contribute to the debate of classifying PSNU as an addictive behavior. We illustrate that specific and general predisposing variables predict both, usage time and severity of symptoms of PSNU, with positive and negative reinforcement processes playing different roles. These roles should compare the impact of these processes on potential problematic online behaviors, but also in the course of behavioral manifestation.

## CRediT authorship contribution statement

**Lena Klein:** Writing – original draft, Visualization, Validation, Project administration, Methodology, Investigation, Formal analysis, Data curation, Conceptualization. **Matthias Brand:** Writing – review & editing, Supervision, Methodology, Funding acquisition, Conceptualization. **Elisa Wegmann:** Writing – review & editing, Supervision, Project administration, Methodology, Funding acquisition, Formal analysis, Data curation, Conceptualization.

## Declaration of competing interest

Given their role as an Editorial Board member Brand M., had no involvement in the peer-review of this article and had no access to information regarding its peer-review. All other authors declare that they have no known competing financial interests or personal relationships that could have appeared to influence the work reported in this paper.

## Data Availability

Data will be made available on request.
