# Supplementary material for: Seeking pleasure or seeking compensation: a dual-pathway model of problematic social networks use
Source: Addict Behav Rep. 2026 Jun 19;24:100720. doi: 10.1016/j.abrep.2026.100720 (PMC13316084; doi:10.1016/j.abrep.2026.100720)
Supplement: Supplementary file 1 — Supplementary material [file mmc1.docx]

# **Supplementary material**

**Personality-related predisposing variable:**

Big Five Inventory-2 (Danner et al., 2019; Soto & John, 2017): Subscale *extraversion*. 12 items. Five-point Likert-scale (1=disagree to 5=strongly agree) and calculated as a mean score. The Cronbach’s alpha was .85. German version was used.

Naughty Nine Scale (Küfner et al., 2015): Subscale *narcissism*.3 items. Nine-point Likert scale (1=strongly disagree to 9=strongly agree). A mean score was used. The Cronbach’s alpha was .88. German version was used.

**Motivational SNS predisposing variable:**

Motives for Using Social Media Scale (MUSM; (Al-Menayes, 2015)). Subscales: *personal utility* (4 items; Cronbach’s alpha: .56), *information seeking* (4 items; Cronbach’s alpha: .58) and *altruism* (2 items, Spearman-Brown: .580). Given that the subscales only consists of a few items, we consider that the reliability is acceptable. For each sub facet a mean score was used. The items were answered on a five-point Likert scale (1=not at all, to 5=exactly). Translated back and forth into German language.

**Loneliness-related SNS predisposing variable:**

short Loneliness Scale (LON; (Gierveld & Tilburg, 2006)): The subscales *emotional loneliness* (3 items; Cronbach’s alpha: .68) and *social loneliness* (3 items; Cronbach’s alpha: .85) of the short Loneliness Scale (LON; (Gierveld & Tilburg, 2006)) were answered by the participants on a scale ranging from 1=yes! To 5=no! and a mean score was calculated for each subscale. Translated back and forth into German language.

**Stress-appraisal & Coping:**

Brief COPE (Carver, 1997; Knoll et al., 2005): Subscales: *denial* (2 items; Spearman-Brown: .614) and *behavioural disengagement* (2 items; Spearman-Brown: .482). Four-point Likert scale from 1=not at all to 4=very likely and a mean score for each subscale. German version was used.

Trier Inventory of Chronic Stress (TICS; (Schulz et al., 2004)): Subscale *chronic stress screening scale* (Cronbach’s alpha: .88). Four-point Likert scale from 1=never to 4=often. Sum scores was used. German version was used.

Temperament and Character Inventory (TCI; (Cloninger et al., 1994; Petrowski et al., 2012)) for assessing self-directedness (44 items; Cronbach’s alpha: .86). Scale from1=yes to 0=no. Sum scores was used. German version was used.

**Positive Reinforcement:**

Experience of Gratification Scale (EGS; (Wegmann et al., 2022)): Subscale: *gratification of needs* (3 items). Five-point Likert scale from 0=never to 4=very often. The Cronbach’s alpha was .66. A mean score was used. German version was used.

Internet Use Expectancies Scale modified for SN use (IUES; (Brand et al., 2014): subscale *positive expectancies* (4 items, Cronbach’s alpha: .82). All answers ranged between 1=completely disagree, to 4=completely agree. A mean score was used. German version was used.

**Negative Reinforcement:**

Experience of Compensation Scale (EGS; (Wegmann et al., 2022)): Subscale: *compensation of needs* (3 items). Five-point Likert scale from 0=never to 4=very often. The Cronbach’s alpha was .71. A mean score was used. German version was used.

Internet Use Expectancies Scale modified for SN use (IUES; (Brand et al., 2014): subscale *avoidance/relief expectancies* (4 items, Cronbach’s alpha: .76). All answers ranged between 1=completely disagree, to 4=completely agree. A mean score was used. German version was used.

**Symptom severity:**

Internet Gaming Disorder Test-10 (IGDT-10; (Király et al., 2017)), modified for social networks use. It consists of ten items, which are answered on a three-point Likert scale, where a score of 0 was assigned if the response was “never” or “sometimes”, and 1 if the participants chose “often”. This procedure was applied to every item. The Cronbach’s alpha was .86. Translated back and forth into German language.

The Assessment of Criteria for Specific Internet-Use Disorders (ACSID-11; (Müller et al., 2022)). Only responses relating to SN use were taken into consideration. The ACSID-11 includes 11 items, which must be answered on two different scales: A four-point Likert scale ranging from 0=never to 3=often, to assess the frequency of the symptoms, and a four-point Likert scale from 0=not intense to 3=intense, for the intensity/severity of the symptoms. A sum score was used for both rating-scales. The frequency scale was used for the analysis. Cronbach’s alpha was .93. German version was used.

AICA-SKI:IBS (Müller et al., 2017) was used to assess problematic usage of the Internet based on the DSM-5 criteria for gaming disorder and has been adopted for PSNU. With this interview, individuals could be classified into certain groups: non-problematic (up to one criterion fulfilled), risky use (> four criteria fulfilled), and PSNU (five to nine criteria fulfilled). Interviews were conducted by trained doctoral students, who received clinical-diagnostic training and regular supervision by experienced clinicians. German version was used.

Al-Menayes, J. J. (2015). Motivations for Using Social Media: An exploratory factor analysis. *International Journal of Psychological Studies*, *7*(1), 43-50. <https://doi.org/10.5539/ijps.v7n1p43>

Brand, M., Laier, C., & Young, K. S. (2014). Internet addiction: Coping styles, expectancies, and treatment implications. *Frontiers in Psychology*, *5*, 1256. <https://doi.org/10.3389/fpsyg.2014.01256>

Carver, C. S. (1997). You want to measure coping but your protocol’ too long: Consider the brief cope. *International Journal of Behavioral Medicine*, *4*(1), 92-100. <https://doi.org/10.1207/s15327558ijbm0401_6>

Cloninger, C. R., Przybeck, T. R., Svrakic, D. M., & Wetzel, R. D. (1994). *The temperament and character inventory (TCI): a guide to its development and use* (1st ed.). Center for Psychobiology of Personality, Washington University.

Danner, D., Rammstedt, B., Bluemke, M., Lechner, C., Berres, S., Knopf, T., Soto, C. J., & John, O. P. (2019). *Das Big Five Inventar 2: Validierung eines Persönlichkeitsinventars zur Erfassung von&nbsp;5&nbsp;Persönlichkeitsdomänen und 15&nbsp;Facetten* (Vol. 65). Hogrefe Verlag. <https://doi.org/10.1026/0012-1924/a000218>

Gierveld, J. D. J., & Tilburg, T. V. (2006). A 6-Item Scale for Overall, Emotional, and Social Loneliness: Confirmatory tests on survey data. *Research on Aging*, *28*(5), 582-598. <https://doi.org/10.1177/0164027506289723>

Király, O., Sleczka, P., Pontes, H. M., Urbán, R., Griffiths, M. D., & Demetrovics, Z. (2017). Validation of the Ten-Item Internet Gaming Disorder Test (IGDT-10) and evaluation of the nine DSM-5 Internet Gaming Disorder criteria. *Addictive Behaviors*, *64*, 253-260. <https://doi.org/https://doi.org/10.1016/j.addbeh.2015.11.005>

Knoll, N., Rieckmann, N., & Schwarzer, R. (2005). Coping as a mediator between personality and stress outcomes: a longitudinal study with cataract surgery patients. *European Journal of Personality*, *19*(3), 229-247. <https://doi.org/10.1002/per.546>

Küfner, A. C. P., Dufner, M., & Back, M. D. (2015). Das Dreckige Dutzend und die Niederträchtigen Neun. *Diagnostica*, *61*(2), 76-91. <https://doi.org/10.1026/0012-1924/a000124>

Müller, K. W., Beutel, M. E., & Wölfling, K. (2017). Klinische Validierung von diagnostischen Merkmalen der Internetsucht. *Suchttherapie*, *18*(S 01), S-10-02. <https://doi.org/10.1055/s-0037-1604540>

Müller, S. M., Wegmann, E., Oelker, A., Stark, R., Müller, A., Montag, C., Wolfling, K., Rumpf, H. J., & Brand, M. (2022). Assessment of Criteria for Specific Internet-use Disorders (ACSID-11): Introduction of a new screening instrument capturing ICD-11 criteria for gaming disorder and other potential Internet-use disorders. *Journal of Behavioral Addictions*, *11*(2), 427-450. <https://doi.org/10.1556/2006.2022.00013>

Petrowski, K., Paul, S., Albani, C., & Brähler, E. (2012). Factor structure and psychometric properties of the trier inventory for chronic stress (TICS) in a representative german sample. *BMC Medical Research Methodology*, *12*(1), 42. <https://doi.org/10.1186/1471-2288-12-42>

Schulz, P., Schlotz, W., & Becker, P. (2004). *Trierer Inventar zum chronischen Stress: TICS; Manual*. Hogrefe, Verlag für Psychologie.

Soto, C. J., & John, O. P. (2017). The next Big Five Inventory (BFI-2): Developing and assessing a hierarchical model with 15 facets to enhance bandwidth, fidelity, and predictive power. *Journal of Personality and Social Psychology*, *113*(1), 117-143. <https://doi.org/10.1037/pspp0000096>

Wegmann, E., Antons, S., & Brand, M. (2022). The experience of gratification and compensation in addictive behaviors: How can these experiences be measured systematically within and across disorders due to addictive behaviors? *Comprehensive Psychiatry*, *117*, 152336. <https://doi.org/10.1016/j.comppsych.2022.152336>
